# Phenotypes of Preterm Birth: A Retrospective Cohort Study from a Tertiary Romanian Centre as a Framework for Future Genomic and Proteomic Research

**DOI:** 10.3390/jcm15051831

**Published:** 2026-02-27

**Authors:** Cristiana-Elena Durdu, Madalina Nicoleta Mitroiu, Bianca Margareta Salmen, Vlad Dima, Adrian Neacsu, Roxana-Elena Bohiltea

**Affiliations:** 1 Doctoral School, Carol Davila University of Medicine and Pharmacy, 050474 Bucharest, Romania; madalina.mitroiu@drd.umfcd.ro (M.N.M.); bianca-margareta.mihai@drd.umfcd.ro (B.M.S.); 2 Department of Neonatology, Carol Davila University of Medicine and Pharmacy, 020956 Bucharest, Romania; 3 Filantropia Clinical Hospital, 011177 Bucharest, Romania; 4 Department of Obstetrics and Gynaecology, Carol Davila University of Medicine and Pharmacy, 050474 Bucharest, Romania; roxana.bohiltea@umfcd.ro; 5 Bucur Maternity, St. John’s Hospital, 012361 Bucharest, Romania; 6 Life Memorial Hospital, 010719 Bucharest, Romania

**Keywords:** genomics, proteomics, preterm birth, prediction tools, clinical phenotypes

## Abstract

**Background/Objectives:** Preterm birth (PTB) is a major global cause of neonatal morbidity and mortality, and its heterogeneous mechanisms limit the development of reliable prediction tools. Recent genomic and proteomic studies have highlighted molecular pathways involving inflammation, extracellular matrix dysfunction, and uterine activation, yet their clinical integration remains limited. Defining distinct clinical phenotypes may facilitate more targeted biomarker research. **Methods:** We performed a retrospective cohort study of singleton spontaneous preterm births (24–36 + 6 weeks) at Filantropia Clinical Hospital, Bucharest (2022–2024). Maternal and neonatal data were extracted from electronic records. Four phenotypes were defined by presentation (preterm premature rupture of membranes—PPROM vs. contractions) and maternal inflammatory status. Statistical comparisons used ANOVA or Kruskal–Wallis tests, Chi-square tests, and logistic regression adjusted for gestational age and birth weight to assess neonatal outcomes. **Results:** Of 585 preterm births, 318 spontaneous singleton cases met inclusion criteria. The cohort was predominantly late preterm, with 85.5% of deliveries occurring between 34 and 36 + 6 weeks’ gestation. Four phenotypes were identified: phenotype 1 inflammatory PPROM (22.3%), phenotype 2 structural PPROM (38.1%), phenotype 3 mixed inflammatory + uterine activation (11.9%), and phenotype 4 uterotonic/endocrine phenotype (19.2%). **Conclusions:** These clinical phenotypes exhibited distinct maternal and neonatal patterns and correspond to mechanisms increasingly supported by genomic and proteomic studies. They may provide a practical framework for integrating clinical and molecular approaches in future PTB research.

## 1. Introduction

According to the World Health Organisation, preterm birth is defined as delivery before 37 completed weeks of gestation [1]. Nevertheless, there is some geographic variation regarding the threshold used to discriminate preterm deliveries from late miscarriages [2]. Preterm birth cases account for 15 million worldwide annually, resulting in an 11% rate of preterm deliveries. Furthermore, preterm birth is the main cause of paediatric deaths, with a mortality rate of 18% for children under the age of 5 years, and 35% for newborns (under 28 days) [3].

Regardless of the mode of delivery (vaginal delivery or caesarean section), preterm birth can occur through several distinct clinical pathways, including preterm premature rupture of membranes (PPROM), spontaneous preterm labour with intact membranes, cervical insufficiency, and medically indicated preterm birth [2,4]. Another way to classify preterm births is by gestational age: roughly 5% of preterm births occur before 28 weeks (extreme prematurity), 15% between 28 and 31 weeks (severe prematurity), 20% at 32–33 weeks (moderate prematurity), and 60–70% between 34 and 36 weeks (late preterm or near term) [2].

Negative outcomes of prematurity include a higher rate of necrotising enterocolitis, sepsis, respiratory conditions (bronchopulmonary dysplasia and respiratory distress syndrome), neurological conditions (intraventricular haemorrhage, hypoxic-ischemic encephalopathy, cerebral palsy, periventricular leukomalacia, seizures), hearing deficiencies, feeding incapabilities, and visual problems [5,6,7].

Developing prediction instruments that can accurately identify high-risk pregnancies at an early gestational age could significantly reduce the consequences of preterm birth. Therefore, close monitoring until birth and risk-lowering treatments could benefit these women. These prediction tools could be particularly useful in low-resource countries where funds have to be directed towards high-risk women with better triaging of cases [8].

Preterm birth, whether spontaneous or iatrogenic, has numerous causes and predisposing variables, hindering the formulation of effective prediction tools [9]. In this context, integrative omics-based research has gained increasing prominence, with genomics, transcriptomics, proteomics, metabolomics, and microbiome studies being applied to better capture the biological heterogeneity of preterm birth and to support clinically meaningful phenotypic stratification [10].

Genomics and proteomics have begun to identify biomarkers linked to inflammation, immunology, and tissue response; however, these advancements necessitate integration into therapeutic practice. This retrospective study analyses the clinical and paraclinical attributes of spontaneous preterm deliveries at Filantropia Clinical Hospital in Bucharest, Romania, from 2022 to 2024. The objective is to identify phenotypes that may enhance future omics-based research.

## 2. Materials and Methods

### 2.1. Study Design

The present study utilises an observational, retrospective design. The records and medical documents of pregnant women who delivered at the Filantropia Clinical Hospital, Bucharest, from January 2022 to December 2024 were analysed to conduct this study. The retrospective data included information such as demographic details, the results of diagnostic tests, the medical histories of the pregnant women, the treatments administered, the obstetrical outcome and other variables relevant to the study’s purpose. Furthermore, we gathered data about neonatal outcomes such as birth weight, Apgar score, length of hospital stay, NICU (Neonatal Intensive Care Unit) admission, duration of NICU stay, resuscitation at birth, need for ventilatory support, results of blood tests, and administered treatments.

The study was conducted in accordance with the Declaration of Helsinki guidelines, and the Ethics Committee of the Filantropia Clinical Hospital, Bucharest, approved the protocol (protocol code 4901/30 May 2025).

### 2.2. Participants

The participants in this study were selected from a total of 585 preterm births recorded at Filantropia Clinical Hospital, Bucharest, between January 2022 and December 2024; of these, 318 pregnant women met the study’s specific inclusion criteria and were included in the final analysis. The inclusion criteria encompassed all types of singleton pregnancies that delivered preterm, defined as delivery between 24 and 37 completed weeks of gestation, during the study period. The exclusion criteria included multiple pregnancies, medically indicated preterm deliveries (severe preeclampsia, severe fetal growth restriction, placental abruption, severe cholestasis, umbilical cord prolapse, placenta accreta spectrum, placenta praevia with bleeding, suspected fetal compromise), and pregnancies with major fetal congenital anomalies or poor-prognosis genetic syndromes.

No restrictions were applied to age or other demographic characteristics of participants. All available information was obtained from the hospital records, including history of medical illness, laboratory tests, and other additional clinical information necessary for the purpose of this study. These demographic and clinical variables were then incorporated for the purpose of analysis and interpretation of the results. All women received routine clinical care in accordance with the usual management of preterm birth, and all procedures related to data were conducted in accordance with applicable ethical and regulatory requirements for medical research. All patient information was kept confidential, and all data were anonymised prior to analysis.

Notably, no biological samples (maternal or neonatal blood, placental tissue, or amniotic fluid) were stored at the time of delivery; therefore, genomic or proteomic analyses could not be performed within this cohort.

### 2.3. Definitions and Clinical Variables

Preterm birth was defined as delivery before 37 completed weeks of gestation as per World Health Organisation criteria [1]. Maternal age at delivery was in years and gestational age in weeks based on first-trimester ultrasound dating and, if unavailable, the last menstrual period. Hypertensive disorders of pregnancy included preeclampsia, gestational hypertension, chronic hypertension, and superimposed preeclampsia based on the International Society for the Study of Hypertension in Pregnancy (ISSHP) 2021 classification [11]. Diabetes included gestational diabetes mellitus and pre-existing diabetes, defined by the American Diabetes Association guidelines [12]. Thrombophilia was defined according to the diagnostic criteria of the American Society of Hematology 2023 guidelines for the management of venous thromboembolism [13].

We defined four mutually exclusive clinical phenotypes according to two dimensions: clinical presentation (PPROM versus painful uterine contractions) and maternal inflammatory activation (present or absent) (Table 1). Maternal inflammatory activation was defined by the presence of at least one of the following criteria: (1) a documented urinary infection during pregnancy; (2) a documented genital infection during pregnancy; (3) a positive vaginal/cervical culture at admission to hospital; (4) a positive urinary culture at admission to hospital; (5) a maternal white cell count greater than 15,000/mm3 at admission; (6) a maternal C-reactive protein level greater than 10 mg/L at admission; or (7) a documented colonization by Group B Streptococcus.

Inflammation was recorded as absent only when all the inflammatory markers available were negative. In order to prevent misclassification caused by the unavailability of laboratory or microbiological data, inflammatory status was only assigned when at least four of the seven criteria listed above were available (Table 1). Cases with insufficient laboratory or microbiological data to allow reliable phenotype assignment were excluded from statistical analyses. No imputation was performed, and all analyses were conducted using a complete-case approach; a total of 27 cases (8.5%) were excluded due to missing data.

This hypothesis-driven phenotypic classification was defined a priori (not previously validated or published) based on two consistently available dimensions: clinical presentation (PPROM vs. uterine contractions) and maternal inflammatory activation. Phenotypes were mutually exclusive, and overlapping conditions such as placental dysfunction were not included in the phenotype definition and therefore did not influence phenotype assignment in this retrospective cohort. Moreover, severe overlapping conditions were excluded from the study.

While subclinical intrauterine inflammation may exist, its detection requires sophisticated or invasive tests, such as amniotic fluid cytokine measurements, PCR for intra-amniotic infection, or other advanced assays, which are not routinely available in clinical practice and may be costly or impractical for large cohorts. Our simple and reproducible phenotypic classification relies on readily available clinical and laboratory markers, ensuring cost-effectiveness and feasibility. Importantly, several genomic and proteomic markers have already been associated in the literature with specific preterm birth phenotypes. By defining clear clinical groups, this framework can guide future studies in selecting the most relevant markers for each phenotype, evaluating their diagnostic or prognostic value, and ultimately, if validated, applying this system in routine clinical practice to optimize biomarker use in preterm birth management.

Cut-off values of ≥15,000/mm^3^ for maternal white blood cell count and ≥10 mg/L for C-reactive protein were adopted to define maternal inflammation, in accordance with thresholds previously used in the literature to identify clinically significant inflammatory activation in preterm birth [14,15].

Statistical analysis included composite maternal and neonatal outcomes. In the maternal composite outcome, there was the presence of hypertensive disorders of pregnancy, presence of diabetes, presence of thrombophilia, conception via In Vitro Fertilisation (IVF), mode of delivery and receipt of treatments. The neonatal composite outcome included total and NICU length of stay, need for resuscitation at birth, need for ventilatory support, need for surfactant and neonatal antibiotic therapy.

### 2.4. Data Collection

Data for the retrospective study were extracted from the electronic medical records of 318 women admitted for preterm birth at Filantropia Clinical Hospital. Demographic characteristics, obstetric and medical history, evolution of pregnancy, clinical examination, laboratory tests, and maternal treatment during the hospital stay were retrieved. Neonatal data, such as birthweight, Apgar score, treatments administered, and early birth outcomes, were also collected. All of the data were managed in compliance with the institution’s policy on medical documentation, in the strictest confidentiality, and data were anonymised. Following data collection, data were entered into an Excel database and underwent a structured cleaning and validation process to identify missing, inconsistent, or implausible values.

### 2.5. Statistical Analysis

Statistical analysis was performed using JASP (version 0.95.4, University of Amsterdam, The Netherlands). We considered a *p* < 0.05 significant, with a confidence of 0.95. Given the exploratory nature of the analysis, no correction for multiple comparisons was applied, and all reported *p*-values represent nominal significance at an alpha level of 0.05. All continuous variables with a Gaussian distribution were reported as mean (±standard deviation), whereas the median (inter-quartile range was used for non-Gaussian distributions. For nominal variables, absolute frequencies (percentages) were reported. To evaluate local and total normality of distribution of continuous variables, we used Q–Q plots and descriptive statistics.

The significance of the difference between groups was evaluated using a one-way ANOVA for Gaussian-distributed variables and a Kruskal–Wallis test for non-Gaussian-distributed variables. For categorical variables, we used a Chi-square test. To evaluate the independent association of phenotypes with neonatal outcomes, we constructed multivariate logistic regression models. To reduce confounding effects, we adjusted for gestational age at delivery and birthweight. Results are expressed as odds ratio (OR) with the 95% confidence interval (CI).

As no single neonatal outcome was pre-specified as a primary endpoint, the analyses were intended to explore potential associations across phenotypic groups. Therefore, the reported *p*-values should be interpreted as hypothesis-generating rather than confirmatory.

## 3. Results

**Figure 1:**
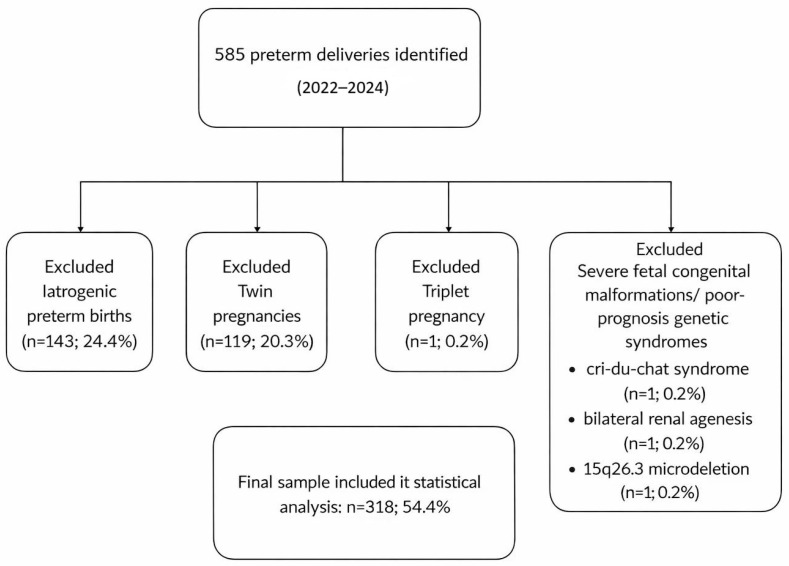
Flowchart illustrating identification, exclusion criteria, and final selection of preterm birth cases (2022–2024).

From 2022 to 2024, Filantropia Clinical Hospital in Bucharest recorded a total of 585 preterm births. Among them, 143 were medically indicated premature deliveries, and 119 were twin pregnancies. Moreover, there was one triplet pregnancy, and three cases involved significant fetal congenital malformations or poor-prognosis genetic disorders (cri-du-chat syndrome, bilateral renal agenesis, and 15q26.3 microdeletion). After applying the exclusion criteria, 318 cases were selected for statistical analysis (Figure 1).

No cases of extreme prematurity were recorded. The sample consisted of 18 severe preterm births (5.7%), 28 moderately preterm births (8.8%) and 272 late preterm births (85.5%). At presentation, 109 women (34.3%) were noted to have only painful uterine contractions, and 209 women (65.7%) had ruptured membranes, with or without painful uterine contractions.

Phenotype 1, which represents the inflammatory PPROM phenotype, included 71 patients (22.3%). Women experienced membrane rupture in the setting of a known infectious process or in the setting of distinctively raised inflammatory biomarkers. The most frequent maternal conditions were diabetes (10 patients, 14.1%) and thrombophilia (7 patients, 9.8%).

Phenotype 2, the structural/membrane-integrity PPROM phenotype, represented 121 patients (38.1%) and was defined by rupture of membranes without inflammatory activation. The most common maternal comorbidities in this group were diabetes (20 patients, 16.5%), hypertension (6 patients, 5.0%), thrombophilia (5 patients, 4.1%) and IVF conception (7 patients, 5.8%).

Phenotype 3 comprised 38 patients (11.9%) and represented the mixed phenotype, defined as activation of both inflammatory and uterine systems. Hypertensive disorders, diabetes, and thrombophilia were common in this group (10.5% each).

Phenotype 4, which represents the uterotonic/endocrine/neuromuscular PPROM phenotype, comprised 61 patients (19.2%) and was characterised by clinical uterine contractions with no evidence of inflammation. Diabetes was the most common maternal condition (10 patients, 16.4%), and hypertensive disorders were also common (5 patients, 8.2%). A separate group of 27 women (8.5%) could not participate in any phenotypic group due to a lack of inflammatory laboratory data and were not included in subsequent subgroup analyses Table 2.

As this was a retrospective study, incomplete inflammatory data were unavoidable and reflect limitations of routine clinical documentation rather than a predefined sampling strategy. Although selection bias cannot be entirely excluded, missing data were not systematically linked to maternal disease severity and represent an inherent constraint of retrospective analyses.

There were modest differences in maternal characteristics among the four phenotypes (Table 3). Maternal age and gestational age were not different between groups (mean range 30.32–31.13 years; *p* = 0.90). Latency from admission to delivery differed significantly between phenotypic groups (*p* < 0.01), with Group 1 having the longest median latency (10.93 h, IQR 3.53–34.86) and Group 4 having the shortest median latency (4.75 h, IQR 2.10–11.53). Maternal WBC at admission differed significantly, with Group 1 accounting for the highest mean WBC levels (13.61 ± 3.84 ×10^3^/L) and Group 4 accounting for the lowest mean WBC levels (11.69 ± 2.81 ×10^3^/L; *p* < 0.01). There was no difference in the proportion of patients with documented prenatal visits (range 85.25–90.91%; *p* = 0.54). Similarly, cesarean delivery rates were comparable across phenotypic groups (43.0–49.2%; *p* = 0.49).

Table 4 shows the frequency of relevant obstetric interventions by phenotypic groups. Gestational steroids, tocolysis, magnesium sulfate for fetal neuroprotection, and maternal antibiotics are listed. There was a significant difference in maternal antibiotic use between groups (*p* < 0.01), and phenotypic Group 1 had the highest use, as would be expected given its inflammatory phenotype. There was a slight difference in corticosteroid use (*p* = 0.08) between groups. Tocolysis and magnesium sulfate showed no significant differences between the four phenotypic groups.

Table 5 displays a comparison of neonatal characteristics and short-term clinical outcomes of neonates from the four phenotypic groups. Group 1 (inflammatory PPROM) had the lowest mean birthweight and the highest proportion of infants requiring NICU admission (53.5%), ventilatory support (28.2%) and neonatal antibiotic treatment (40.8%). Groups 2 and 4 had intermediate clinical severity, whilst Group 3 (mixed inflammatory + uterine activation) had a higher percentage of infants in the NICU (47.4%), although with relatively similar neonatal Apgar scores and inflammatory markers. There were no differences between groups for neonatal Apgar scores, inflammatory markers (WBC, CRP, procalcitonin), duration of NICU stay, resuscitation at birth and surfactant requirement.

Unadjusted and adjusted odds ratios (ORs) with 95% confidence intervals for neonatal outcomes in four phenotypic groups (Group 1 as the reference group) are listed in Table 6. With unadjusted ORs, we present the crude association between group membership and each neonatal outcome. Adjusted ORs were estimated by adjusting for gestational age and birth weight to assess whether there are group differences in neonatal outcomes after taking into account these neonatal characteristics. Outcomes were admission to the neonatal intensive care unit (NICU), ventilatory support, antibiotic treatment in the neonatal period, and extended length of stay (>14 days).

## 4. Discussion

This study reports a systematic clinical phenotyping of sPTB in a large Romanian tertiary centre and a four-phenotype classification that manifests distinct maternal phenotypes and aetiological pathophysiological profiles. This classification distinguishes PPROM-related and contraction-related mechanisms as well as the inflammation level at presentation, and it allows us to identify clinically relevant subgroups with different maternal characteristics and clinical outcomes. While numerous differences emerged in the unadjusted analyses (e.g., NICU admission, length of stay > 14 days, antibiotic use), most of the associations did not persist after adjusting for gestational age and birthweight.

Our classification converges on conceptual frameworks already available in the literature that describe the aetiological heterogeneity of preterm birth. A number of pathogenic pathways have been identified as pivotal elements of mechanisms of preterm birth, i.e., infection/inflammation, uterine overdistension, decidual haemorrhage, cervical insufficiency, and endocrine- or stress-mediated activation [2,9].

### 4.1. Clinical Phenotyping Models

#### 4.1.1. PPROM-Related Mechanism

In the current study, the two phenotypic groups identified are complementary in providing insight into the biological heterogeneity of PPROM. Group 1—the “Inflammatory PPROM” phenotype—reflects infection/inflammation-driven pathways described previously in studies of amniotic fluid, chorion and decidua, where microbial invasion and increased inflammatory signalling play a key role in the weakening and rupture of membranes [16]. In contrast, Group 2—“Structural/membrane integrity PPROM”—identifies a distinct biological profile with predominant changes in extracellular matrix integrity and collagen remodelling, suggesting a more mechanical pathway of failure.

The preterm premature rupture of the membranes remains a major cause of preterm birth, responsible for up to 3% of all pregnancies worldwide [17,18]. A number of clinical factors have been associated with increased risk for PPROM. The history of PPROM is one of the strongest predictors, with a recurrence rate of nearly 14% [19]. Additionally, the presence of pathogens in the vaginal or cervical samples may make a woman susceptible to ascending infection, histologic chorioamnionitis, and eventually rupture of the membranes [20]. The development of intrauterine infection in a scenario in which the membranes rupture prior to the onset of labour can result in significant maternal and fetal morbidity, ranging from fetal death to severe maternal sepsis [21]. Importantly, intrauterine infection is, in most cases, a secondary event. It follows the loss of integrity of the membranes as opposed to being the primary cause. Equally suggestive is the fact that some cases of PPROM occur in the absence of microbial invasion. In such cases, the fragility may be due to increased proinflammatory cytokine activity or structural weaknesses in the components of the membranes [22].

These mechanistic variations have significant implications for prediction, risk stratification and targeted management. Proteomic and genomic advances have highlighted the therapeutic potential of molecular biomarkers that have a dual clinical role. Biomarkers identified in patients with PPROM may be used to predict delivery timing and the risk of imminent labour, facilitating earlier therapeutic interventions to prolong latency or improve neonatal outcomes. Alternatively, particular molecular signatures occurring before rupture of the membrane may enhance the prediction of women at high risk of PPROM to facilitate prophylaxis with targeted surveillance, progesterone treatment or cervical support [8,10].

Proteomic studies have identified a variety of candidate biomarkers, comprising amniotic fluid, cervicovaginal fluid, blood and/or placental biomarkers, that have consistently been found to be associated with PPROM.

Consequently, a substantial body of proteomic work supports the hypothesis that PPROM is likely to be heterogeneous, occurring through distinct biological pathways, most commonly inflammation and membrane structural weakening. Studies assessing short-term predictors of delivery in women with established PPROM correspond clearly to these mechanistic domains [19,22].

Biomarkers associated with imminent delivery were repeatedly implicated in inflammatory processes, indicating neutrophil recruitment and cytokine signalling as well as systemic immune activation. Kim et al. described increased amniotic fluid IL-8, lipocalin-2, MMP-9, and S100 A8/A9 levels as predictive of delivery within 14 days [23]. Ronzoni et al. showed that increased maternal serum endotoxin activity was predictive of delivery within seven days [24]. Similarly, Ryu et al. showed that high maternal CRP, lipid peroxides, and protein carbonyls and low antioxidant capacity were found to be major predictors of delivery in <3 days, therefore supporting the involvement of inflammatory and oxidative stress pathways [25]. Rahkonen et al. demonstrated an inflammatory signature through cervical fluid where MMP-8 was found to be higher in PPROM and lower in women who had a spontaneous preterm birth [26].

Contrarily, we found that the structural/extracellular matrix degradation biomarker phenotype represents a phenotype in which preterm rupture is mainly due to loss of connective tissue rather than overt inflammation. Gezer et al. found that high levels of cervicovaginal urea and creatinine predicted delivery within 48 h and were supportive of PPROM diagnosis by suggesting increased membrane permeability and amniochorion structural compromise [27]. Proteomic analysis of fetal membranes also showed differential expression of proteins implicated in extracellular matrix remodelling (COL4A1, LAMA2, LMNB1/2, FBLN2, CSRP1), proteolysis (CTSG, ELANE, MMP-9), and energy metabolism pathways (PKM, ADPGK, ATP6AP1), indicating that extracellular matrix degradation and energy metabolism exhaustion are additional, non-inflammatory pathways contributing to membrane failure [28].

Genomic evidence for a unique biological architecture for PPROM: predominantly extracellular matrix instability, coagulation, and localised inflammatory activation. Genomic variants associated frequently with membrane rupture susceptibility are matrix remodelling genes such as TIMP2 (rs2277698) and (rs1882435), consistent with the structural degradation mechanism delineated by proteomic studies [29,30,31]. Polymorphisms in TNF-related pathways, such as TNFRSF6 (tumour necrosis factor receptor superfamily member 6 or Fas gene), have also been reported in several studies of PPROM, implying that abnormal apoptotic signalling may be involved in premature weakening of the membranes [32]. PPROM susceptibility has also been associated with variants in toll-like receptors that govern microbial recognition and localised inflammatory responses, as reported by Ramos et al. and Romero et al. [9,33]. In addition, systems-level genomic investigations suggest that PPROM-specific gene networks may be regulated by ESR2 and STAT1, implicating hormonal and immune-mediated mechanisms. In summary, current evidence shows that the underlying genomic background of PPROM encompasses collagen metabolism, matrix degradation, haematologic/coagulation dysregulation and localised inflammatory activation, and that it defines a phenotype distinct from contraction-driven preterm labour [34].

From an omics perspective, these two PPROM-related phenotypes provide a clear framework for targeted molecular investigation. Inflammatory PPROM may be explored using proteomic and transcriptomic profiling of amniotic fluid, cervicovaginal fluid, or maternal blood collected at admission, focusing on immune activation and cytokine signalling pathways. In contrast, the structural/membrane integrity PPROM phenotype may benefit from proteomic analysis of fetal membranes or cervicovaginal fluid, targeting extracellular matrix remodelling, collagen turnover, and metabolic exhaustion pathways. Such phenotype-driven sampling strategies may reduce biological heterogeneity and enhance biomarker discovery in future omics-based studies.

#### 4.1.2. Contraction-Related Mechanisms

Phenotypic Group 3—“Mixed inflammatory + uterine activation” phenotype is a hybrid biological pathway in which mild inflammatory signalling is accompanied by early activation of the uterine contractile machinery. This may indicate that even subclinical inflammation decreases the myometrial activation threshold and initiates the process of progressing from membrane weakening to active labour. Phenotypic group 4—“Uterotonic endocrine/neuromuscular” phenotype—represents a more contractility-directed biological process that involves endocrine, neuromuscular and calcium-handling pathways that directly increase myometrial excitability and induce coordinated uterine contractions. As in the inflammation-dominated phenotype, this pathway is likely to indicate a process of labour that is less related to infection-mediated pathways and more to uterotonic intrinsic signalling and the uterus’s neuromuscular readiness.

Several inflammation-related biomarkers, including calgranulin C, annexin A5, α-1-antitrypsin precursor and kininogen, were consistently upregulated in cervical or vaginal fluids of women who went on to progress from preterm labor to preterm birth, suggesting leukocyte recruitment, cytokine signaling and tissue remodeling, whereas S100 calcium-binding protein A7, histone H2B, peptidyl-prolyl cis-trans isomerase A, L-lactate dehydrogenase A chain, histone H1.2, cystatin B and histone were markedly downregulated in women who delivered preterm, implying a suppression of nuclear stability and cytoskeletal homeostasis at the time of inflammatory uterine activation [35].

In addition, endocrine, neuromuscular and metabolic pathways have been implicated in the activation of contractions as revealed by functional analyses that demonstrate differential expression of proteins associated with vitamin D metabolism, plasminogen activation, blood coagulation, angiogenesis and receptor/signal-transducer regulation, which suggests coordinated alterations in hormonal responsiveness, calcium homeostasis and excitability of the myometrium. First-trimester proteomic signatures for later spontaneous preterm birth, including vitamin D-binding protein, apolipoprotein A-I and α-1-antitrypsin, provide further evidence of an endocrine–metabolic susceptibility that may precede clinical onset of labour [36].

Spontaneous preterm labour with intact membranes (sPTB) is associated with a biological profile characterised by an inflammatory regulatory profile, uterine activation, and a neuroendocrine signalling signature, according to genomic data. Multiple SNPs identified in European-derived populations, including EBF1 and NFKB1, underscore the importance of inflammatory transcriptional control in determining gestational length [37,38]. Importantly, genome-wide analyses of transcriptional regulators suggest that the genetic variant rs3820282 at the WNT4 locus may be functionally linked to regulatory activity, including ESR1 binding, and could influence WNT4 expression, a gene involved in uterine development and hormonal signaling, thereby supporting a potential mechanistic link to timing of labour onset [37]. Targeted analysis identified several TNF pathway genes associated with sPTB, further underscoring the role of systemic immune activation in uterine contractility [39,40,41]. Integrated network analysis reveals that sPTB is generated by an autoimmune-hormonal axis, with upstream regulators including NR3C1 (glucocorticoid receptor), PPARG and IRF3, and these susceptibility profiles are distinct from those of PPROM. Altogether, this genomic evidence provides a framework in which sPTB is largely determined by a hormonal, innate immunity, and neuromuscular activation signature, supporting a contraction-dominant versus structural membrane-failure hypothesis [34,42].

For contraction-dominant phenotypes, future omics approaches could focus on transcriptomic and proteomic profiling of maternal blood, myometrial tissue (where available), or cervicovaginal fluid collected at the onset of symptoms. These analyses may target hormonal signalling, calcium-handling pathways, neuromuscular excitability, and inflammatory transcriptional regulators. Longitudinal sampling prior to and during the onset of contractions may be particularly informative for identifying molecular signatures associated with uterine activation.

### 4.2. Maternal and Neonatal Outcomes Across Phenotypes

Distinct clinical patterns emerged in the four phenotypic groups identified in this study. Maternal comorbidities were unevenly distributed, consistent with the biological differences suggested by the clinical phenotypes. Group 1 (inflammatory PPROM) was characterised by moderate rates of metabolic and vascular comorbidities, specifically diabetes (14.1%) and thrombophilia (9.8%), as previously reported in relation to premature membrane weakening and systemic inflammation and metabolic dysfunction [43,44,45]. Group 2 was characterised by the most frequent rates of diabetes (16.5%) and similar prevalence of hypertensive disease and thrombophilia, indicating a phenotype with chronic metabolic or vascular stress that might compromise extracellular matrix integrity without the overt presence of inflammation [46,47,48,49]. Group 3 had similar rates of hypertensive disease and thrombophilia (10.5% each), suggesting that both inflammatory and contractile pathways might be stimulated in women with underlying vascular vulnerability. Indeed, as reported, endothelial dysfunction and microvascular lesions of the placenta, typical of thrombophilia, instigate local hypoxia, oxidative stress, and cytokine release that stimulate prostaglandin synthesis and uterine activation [50]. Likewise, hypertensive pregnancy disorders manifest as systemic endothelial activation and increased levels of inflammatory mediators, mechanisms implicated previously in spontaneous preterm labour independent of membrane status [51].

Such findings are entirely in keeping with the “vascular obstetrical syndrome” model, which postulates that vascular dysfunction predisposes to a simultaneous inflammatory and uterotonic response, closely resembling the mixed phenotype apparent in phenotypic Group 3.

Group 4 had the highest prevalence of hypertensive disorders, at 16.4%, which is consistent with a mechanistic link between vascular dysfunction and uterine excitability. Hypertensive disease induces endothelial activation, oxidative stress, and altered prostaglandin metabolism-all key biological processes that enhance myometrial sensitivity and promote spontaneous uterine contractions [52]. Also, increased oxytocin receptor expression and exaggerated myometrial calcium signalling have been reported in hypertensive pregnancies, further supporting an endocrine–vascular interaction that favours a pro-contractile uterine environment [53].

Overall, the distribution of comorbidities across clinical phenotypes underscores the notion that PPROM and preterm labour are heterogeneous syndromes with different maternal risk profiles.

Maternal age was similar among all four groups, while gestational age at delivery, latency from admission to delivery and maternal WBC differed significantly (*p* < 0.01). In keeping with the well-recognised observation that gestational age is the dominant determinant of early neonatal morbidity and mortality, even small differences of approximately 1 week between groups are likely to be clinically relevant [54].

The inflammatory PPROM Group 1 delivered at the lowest mean gestational age and had both the longest latency from admission to delivery and the highest maternal WBC count. This pattern is consistent with data showing that intra-amniotic infection/inflammation is associated with earlier preterm delivery, prolonged latency after membrane rupture at lower gestational ages, and maternal leukocytosis as part of the clinical picture of chorioamnionitis [54,55,56,57].

By contrast, the structural/membrane-integrity group and the two contraction-related groups delivered at more advanced gestations with progressively shorter latency intervals and lower WBC values in a trajectory compatible with non-infectious mechanisms of preterm parturition dominated by mechanical membrane weakness or uterine activation rather than overt inflammation. This interpretation agrees with conceptual models that distinguish infection-driven PPROM from “uterotonic” or endocrine/neuromuscular pathways of spontaneous preterm birth [9].

Phenotypic group differences in the proportion of monitored pregnancies and cesarian deliveries did not differ significantly, indicating that the phenotypic differences reported here are unlikely to be explained simply by variations in antenatal surveillance or obstetric management within this cohort.

Antenatal corticosteroid use was relatively similar across groups (14.0–28.2%, *p* = 0.08). This pattern is consistent with current European guidelines, which recommend offering a single course of corticosteroids to all women at risk of preterm birth between about 24–34 weeks’ gestation, irrespective of the underlying etiology of preterm birth [58]. Thus, in our cohort, steroid administration appears to have been driven mainly by gestational age and imminent risk of delivery rather than by phenotype.

Tocolysis rates were also similar across phenotypic groups, from (9.1–19.7%, *p* = 0.17). Current recommendations stress that tocolytic agents are to be employed judiciously, primarily to buy 48 h to complete corticosteroid treatment and/or in-utero transfer, and that their application is clinically based on criteria of cervical change, the absence of contraindications, rather than on putative biological pathways leading to preterm labour [59]. The absence of a significant between-group difference is thus in line with current practice recommendations.

Use of magnesium sulfate for fetal neuroprotection was low in all groups (0–2.8%, *p* = 0.25). International recommendations support intravenous magnesium sulfate when early preterm birth (generally <32 weeks) is imminent, to reduce the risk of cerebral palsy and severe motor impairment in surviving infants [60]. The low use of magnesium sulfate in our cohort most probably reflects the clinical profile of our population, as we had no cases of extreme prematurity and only a small proportion of severe preterm births—situations in which neuroprotective magnesium sulfate is usually indicated.

By contrast, there was a significant difference in maternal antibiotic use in phenotypic groups (26.2–74.6%, *p* < 0.01), with Groups 1 and 2 having the highest exposure to antibiotics (74.6% and 62.0%, respectively). These results are consistent with evidence and guidelines that suggest prophylactic antibiotics should be given to women diagnosed with PPROM to prolong latency and reduce infectious morbidity of mothers and neonates [61,62]. Antibiotic use was significantly lower in Group 4 (uterotonic/endocrine phenotype) and Group 3 (mixed inflammatory + uterine activation) at 42.1% and 26.2%, respectively. This is consistent with recommendations that routine antibiotic therapy should be avoided in women diagnosed with idiopathic preterm labor with intact membranes, as no benefits were identified and the potential for harm in the ORACLE II study and subsequent analyses [63,64].

This study demonstrates that the important determinant of neonatal outcomes is more likely fetal maturity at the time of delivery rather than membership in a particular phenotypic group, as noted in a previous study [65]. In our study, we observed no differences in neonatal inflammatory markers such as CRP and procalcitonin between the two groups, which would provide additional support to this observation. Notably, phenotypic Group 1 had the highest incidence of NICU admission (53.5%) and neonatal ventilatory support (28.2%), but no differences reached significance, which again supports the literature that gestational age is more indicative of neonatal outcomes than membership in a particular group [66,67,68].

The unadjusted and adjusted odds ratios for neonatal outcomes across phenotypes identify an interesting pattern. After adjustment for gestational age and birth weight, the risk of ventilatory support was borderline significant for Group 2 (adjusted OR 3.06; 95% CI 1.00–9.37; *p* = 0.05) versus Group 1 (reference). This result is counterintuitive because Group 2 is defined by a structural/membrane integrity pathway rather than overt inflammatory activation, yet it carries an approximately three-fold increased risk of neonatal ventilatory requirement. A plausible biological explanation is that although inflammation can make the uterus contract and trigger delivery, it also accelerates fetal lung maturation, which reduces the need for ventilatory support in the newborns of subjects with more inflammatory phenotypes. Both experimental and clinical studies have proven that intrauterine inflammation accelerates surfactant production and improves lung compliance, hence reducing the incidence of respiratory distress syndrome (RDS), even in preterm infants [69,70,71].

This observation may further support, but does not confirm, the hypothesis that latency from membrane rupture to delivery differs between inflammatory and non-inflammatory phenotypes, as latency was significantly longer in the inflammatory phenotype compared with the non-inflammatory phenotype (mean 83.37 h vs. 24.30 h, *p* = 0.01), suggesting more prolonged intrauterine exposure in the presence of inflammatory activation. The shorter latency observed in the non-inflammatory group may reflect a more abrupt transition to delivery without prior inflammatory stimulation, which may be associated with relatively less advanced biological pulmonary maturation despite similar gestational age at birth. In addition, antenatal corticosteroid administration represents a potential confounder; however, in our institution, corticosteroid therapy follows a standardized protocol strictly based on gestational age (all pregnancies < 34 weeks receive dexamethasone), independent of phenotypic classification. While gestational age was included in the adjusted models, residual confounding related to the timing and completeness of antenatal corticosteroid exposure cannot be excluded, given the retrospective nature of the data.

However, given the exploratory design of the study and the multiple comparisons performed across neonatal outcomes, this association may reflect a type I error and should therefore be interpreted as hypothesis-generating, and larger cohorts are needed to confirm the finding.

### 4.3. Strengths and Limitations

The strengths of this study include the fact that we gathered data from only one tertiary centre with standardised therapeutic protocols, thereby ensuring that data collection and case management were uniform. The proposed phenotypic classification enabled a structured clinical characterization of preterm birth cases, providing a multidimensional descriptive framework based on established obstetric presentations. The use of comprehensive clinical, laboratory and microbiological data and rigorous statistical methods, such as multivariable logistic regression adjusted for gestational age and birthweight, facilitated assessment of the associations observed under appropriate analytic conditions. Further studies are now able to use the framework developed here, but with immediate benefit to future omics-based work by mapping genomic and proteomic signatures within a clinically focused conceptual framework.

This study also has some limitations. First of all, it was conducted in a retrospective manner, associating inherent risks specifically related to information bias and limiting causality. Some of the biomarker information collected from the patient’s blood test results, such as white blood cell (WBC) count and C-reactive protein (CRP) levels, was unavailable for a subset of patients. This led to difficulty in accurately comparing the levels of inflammation between the different groups. Furthermore, the lack of placental histopathology and cytokines in the amniotic fluid hinders providing a more detailed overview of the biological processes occurring during preterm birth. Additionally, this is a single-centre study, comprising patients with similar demographic characteristics; therefore, the results of this study may not apply to other populations.

The predominance of late preterm deliveries (34–36+6 weeks) in our cohort limits the direct extrapolation of neonatal outcome patterns to populations with higher rates of extreme prematurity. While the underlying biological pathways defining our phenotypes are likely relevant across gestational ages, their relative impact on neonatal morbidity may differ in settings where early preterm birth and neonatal mortality are more prevalent. External validation in more heterogeneous populations, including low-resource settings, is therefore warranted.

Lastly, since no samples were stored for later analysis, it was impossible to validate certain genomic or proteomic markers that may contribute to preterm birth in this population.

### 4.4. Clinical Implications of Phenotype-Based Classification

**Figure 2:**
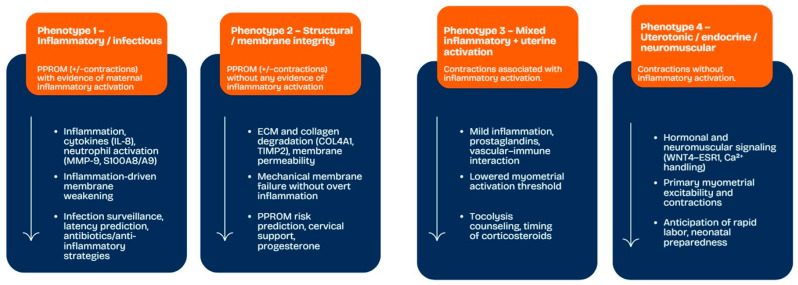
Conceptual framework of spontaneous preterm birth phenotypes and their underlying biological pathways.

Phenotype-based classification of spontaneous preterm birth may offer practical advantages for clinical risk stratification, patient counseling, and neonatal management. By distinguishing inflammation-dominant, structural membrane–related, and contraction-driven pathways, this framework allows clinicians to better contextualize the expected clinical course, including latency to delivery and potential neonatal risks (Figure 2).

From a preventive perspective, phenotype identification may support more personalized strategies. For example, women with inflammatory phenotypes may benefit from intensified infection surveillance and targeted antimicrobial or anti-inflammatory interventions, whereas those with structural or contraction-dominant phenotypes may be more suitable candidates for progesterone therapy, cervical support, or closer uterine activity monitoring. Similarly, recognition of contraction-driven phenotypes may guide anticipatory counseling regarding tocolysis efficacy and timing of antenatal corticosteroid administration.

At the neonatal level, phenotype-informed stratification may improve preparedness for respiratory support, infection surveillance, and NICU resource allocation, even when gestational age at delivery is similar. Overall, integrating clinical phenotyping into obstetric care pathways may represent a first step toward personalized prevention and management of preterm birth, and provides a clinically meaningful foundation for future integration with omics-based risk prediction models.

Prospective validation of this framework would ultimately depend on dedicated prospective studies incorporating standardized, multi-compartment biobanking at the time of clinical presentation. In such studies, maternal peripheral blood (for genomic, transcriptomic, and proteomic profiling), urine (metabolomics), vaginal or cervical secretions (microbiome and local inflammatory mediators), and, when clinically indicated, amniotic fluid could be collected prior to therapeutic interventions. Systematic placental tissue sampling immediately after delivery, using standardized fixation or cryopreservation protocols, would further enable exploratory correlations between clinical phenotypes and tissue-level molecular signatures. Importantly, this proposed strategy represents a conceptual roadmap for future research rather than an extension of the current retrospective dataset.

Although the pathogenic pathways underlying spontaneous preterm birth have been previously described, their operationalization into a structured, mutually exclusive clinical classification remains limited in routine obstetric practice. By applying a reproducible phenotypic framework within a well-characterized tertiary cohort, our study translates established biological concepts into a clinically usable stratification model. This approach reduces heterogeneity within preterm birth populations and provides a practical bridge between bedside clinical phenotyping and future, prospectively designed molecular studies. In this context, the value of the present work lies not in identifying a novel mechanism but in establishing a structured platform upon which subsequent genomic and proteomic investigations may be systematically built.

## 5. Conclusions

This study demonstrates that identifying clinically relevant subtypes of spontaneous preterm birth provides a good foundation for future research studies focused on understanding the molecular basis of preterm birth. The phenotyping system identified structural, inflammatory, endocrine, and mixed forms of preterm birth and, therefore, allows researchers to focus their efforts on specific groups rather than trying to understand the entire population. This can lead to a reduction in heterogeneity among samples selected for multi-omic analyses, increased biological relevance of those analyses, and the development of precision obstetrics approaches to prevent preterm birth. Furthermore, the identification of clinically distinct pathways for preterm birth creates opportunities for the identification of potential therapeutic targets for the prevention of preterm birth. Therefore, future studies on spontaneous preterm birth should include both clinically defined subtypes and the collection of multi-omic data, such as proteomics, genomics, transcriptomics, and metabolomics, fostering identification of specific biological pathways involved in each subtype of preterm birth. Additionally, by collecting group-specific biological samples, investigators will obtain pathway-level insights into the biology of each subtype of preterm birth and develop better prediction tools based on biomarkers. Using machine learning algorithms to combine clinical data and omics data can further improve the accuracy of subtyping spontaneous preterm birth and develop more personalised preventive strategies. To our knowledge, this represents the first structured phenotypic classification of spontaneous preterm birth conducted in a Romanian tertiary centre, providing an essential foundation for future multi-omic research initiatives within this population and facilitating alignment with international precision obstetrics efforts.

## Figures and Tables

**Table 1:** Operational definitions of the clinical phenotypes based on clinical presentation (PPROM vs. contractions) and the presence or absence of maternal inflammatory activation.

Phenotype	Definition
**Phenotype 1**—Inflammatory/infectious	PPROM (+/−contractions) with evidence of maternal inflammatory activation, defined by infections documented during pregnancy and/or laboratory/microbiological findings at presentation.
**Phenotype 2**—Structural/membrane integrity	PPROM (+/−contractions) without any evidence of inflammatory activation.
**Phenotype 3**—Mixed inflammatory + uterine activation	Contractions associated with inflammatory activation.
**Phenotype 4**—Uterotonic/endocrine/neuromuscular	Contractions without inflammatory activation.

**Table 2:** Distribution of the four clinical phenotypes and associated maternal conditions.

Phenotype	No. (%)	Most Frequent Associated Maternal Conditions (%)
Phenotype 1—Inflammatory PPROM	71 (22.3%)	Hypertensive disorders: 3 (4.2%) • Diabetes: 10 (14.1%) • Thrombophilia: 7 (9.8%) • IVF conception: 1 (1.4%)
Phenotype 2—Structural/membrane integrity PPROM	121 (38.1%)	Hypertensive disorders: 6 (5.0%) • Diabetes: 20 (16.5%) • Thrombophilia: 5 (4.1%) • IVF conception: 7 (5.8%)
Phenotype 3—Mixed inflammatory + uterine activation	38 (12.0%)	Hypertensive disorders: 4 (10.5%) • Diabetes: 4 (10.5%) • Thrombophilia: 4 (10.5%) • IVF conception: 4 (10.5%)
Phenotype 4—Uterotonic/endocrine phenotype	61 (19.2%)	Hypertensive disorders: 5 (8.2%) • Diabetes: 10 (16.4%) • Thrombophilia: 3 (4.9%) • IVF conception: 0 (0%)
No data	27 (8.5%)	

**Table 3:** Maternal and obstetric characteristics across the four clinical phenotypes.

	Mean (SD)		Median (IQR)			
Phenotype Group	Maternal Age, Years	Maternal WBC at Admission, ×10^3^/L	Gestational Age at Delivery, Weeks	Latency Admission-to-Delivery, Hours	Monitored Pregnancy, %	Cesarean Section, %
1	30.32 ± 5.83	13.61 ± 3.84	36.00 (33.43–36.57)	10.93 (3.53–34.86)	90.14	47.9
2	30.64 ± 5.66	12.01 ± 3.49	36.14 (35–36.57)	7.12 (3.58–15.45)	90.91	43.0
3	31.13 ± 5.82	12.97 ± 3.25	36.00 (35.32–36.50)	6.84 (2.60–18.13)	86.84	44.7
4	30.39 ± 5.70	11.69 ± 2.81	36.29 (35.71–36.57)	4.75 (2.10–11.53)	85.25	49.2
*p* value	0.9	<0.01	0.07	<0.01	0.54	0.49

**Table 4:** Distribution of obstetric interventions across phenotype groups.

Treatment	Phenotypic Group 1 (*n* = 71)	Phenotypic Group 2 (*n* = 121)	Phenotypic Group 3 (*n* = 38)	Phenotypic Group 4 (*n* = 61)	*p*-Value
Corticosteroid therapy, *n* (%)	20 (28.2)	17 (14.0)	6 (15.8)	9 (14.8)	0.08
Tocolysis, *n* (%)	14 (19.7)	11 (9.1)	7 (18.4)	8 (13.1)	0.17
Magnesium sulfate, *n* (%)	2 (2.8)	0 (0)	0 (0)	1 (1.6)	0.25
Maternal antibiotics, *n* (%)	53 (74.6)	75 (62.0)	16 (42.1)	16 (26.2)	<0.01

**Table 5:** Neonatal outcomes at birth and during hospitalisation across the four phenotypic groups.

Characteristic	Phenotypic Group 1 (*n* = 71)	Phenotypic Group 2 (*n* = 121)	Phenotypic Group 3 (*n* = 38)	Phenotypic Group 4 (*n* = 61)	*p*-Value
Birth weight (g), mean (SD)	2463.38 ± 540.04	2719.01 ± 358.02	2661.84 ± 474.40	2650.00 ± 498.50	<0.01
Apgar score, 1 min, median (IQR)	8 (7–9)	8 (8–9)	8 (7.25–9)	8 (8–9)	0.9
Apgar score, 5 min, median (IQR)	9 (8–9)	9 (8–9)	9 (8–9)	9 (8–9)	0.13
Neonatal WBC at admission (×10^3^/L), mean (SD)	14.14 ± 5.20	14.05 ± 4.22	15.12 ± 6.05	15.82 ± 6.43	0.18
Neonatal CRP at admission (mg/L), median (IQR)	0.88 (0.42–2.23)	1.05 (0.56–1.96)	1.2 (0.54–4.42)	1.1 (0.59–2.33)	0.69
Neonatal procalcitonin (ng/mL), median (IQR)	7.81 (2.73–16.61)	6.41 (2.1–12.46)	3.01 (0.23–10.29)	3.18 (1.53–10)	0.21
Length of stay (days), median (IQR)	6 (3–12.5)	4 (3–8)	4 (3–6.75)	4 (3–7)	0.14
NICU stay (days), median (IQR)	1 (0–5)	0 (0–2)	0 (0–2)	0 (0–2)	0.10
Resuscitation at birth, %	37.7	25.8	21.1	23.0	0.16
NICU admission, %	53.5	38.8	47.4	44.3	0.26
Ventilatory support, %	28.2	21.5	21.1	18.0	0.55
Surfactant needed, %	4.2	0	5.3	3.3	0.14
Antibiotics required, %	40.8	24.8	26.3	23.0	0.07

**Table 6:** Unadjusted and adjusted odds ratios for neonatal outcomes according to phenotypic groups.

Outcome	Phenotypic Group	Unadjusted OR (95% CI)	*p*	Adjusted OR * (95% CI)	*P*
NICU admission	1	1.00 (reference)	—	1.00 (reference)	—
	2	0.55 (0.30–1.00)	0.05	0.86 (0.72–1.84)	0.73
	3	0.78 (0.35–1.72)	0.54	1.41 (0.47–4.19)	0.54
	4	0.69 (0.35–1.37)	0.29	1.73 (0.67–4.48)	0.26
Ventilatory support	1	1.00 (reference)	—	1.00 (reference)	—
	2	0.41 (0.10–1.37)	0.20	3.06 (1.00–9.37)	0.05
	3	1.21 (0.43–3.37)	0.71	2.21 (0.49–10.01)	0.31
	4	1.24 (0.55–2.79)	0.59	2.46 (0.63–9.69)	0.20
Neonatal antibiotics	1	1.00 (reference)	—	1.00 (reference)	—
	2	0.48 (0.25–0.89)	0.02	0.86 (0.43–1.11)	0.73
	3	1.20 (0.45–3.23)	0.70	0.79 (0.22–1.23)	0.69
	4	0.43 (0.20–0.92)	0.03	0.86 (0.20–0.92)	0.78
Length of stay > 14 days	1	1.00 (reference)	—	1.00 (reference)	—
	2	0.33 (0.14–0.78)	0.01	4.06 (0.14–23.15)	0.11
	3	1.32 (0.48–3.55)	0.23	2.73 (0.15–49.88)	0.50
	4	0.36 (0.12–1.08)	0.07	0.57 (0.12–1.08)	0.71

* Adjusted OR for gestational age and birth weight.

## Data Availability

The original contributions presented in this study are included in the article. Further inquiries can be directed to the corresponding author(s).

## Abbreviations

The following abbreviations are used in this manuscript:

PTB	Preterm birth
PPROM	Preterm premature rupture of the membranes
ANOVA	Analysis of variance
WBC	White blood cell count
NICU	Neonatal Intensive Care Unit
IIOC	Incompetence of the internal os of the cervix
IVF	In vitro fertilisation
CRP	C-reactive protein
SD	Standard deviation
IQR	Interquartile range
OR	Odds ratio
ECM	Extracellular matrix
MMP	Matrix metalloproteinase
IL	Interleukin
S100 A8/A9	Calprotectin
COL4A1	Collagen Type IV Alpha-1 Chain
LAMA2	Laminin Subunit Alpha-2
LMNB1/2	Lamin B1/2
FBLN2	Fibulin-2
CSRP1	Cysteine- and Glycine-Rich Protein 1
CTSG	Cathepsin G
ELANE	Elastase, Neutrophil Expressed
PKM	Pyruvate Kinase, Muscle
ADPGK	Adenosine Diphosphate Glucokinase
ATP6AP1	Adenosine Triphosphase H^+^ Transporting Accessory Protein 1
TIMP2	Tissue Inhibitor of Metalloproteinases 2
TNF	Tumour necrosis factor
TNFRSF6	Tumour necrosis factor receptor superfamily member 6 (Fas receptor)
ESR2	Estrogen receptor 2
STAT1	Signal Transducer and Activator of Transcription 1
SNP	Single Nucleotide Polymorphism
EBF1	Early B-Cell Factor 1
NFKB1	Nuclear Factor Kappa B Subunit 1
WNT4	Wingless-Type Mouse Mammary Tumor Virus Integration Site Family, Member 4
NR3C1	Nuclear Receptor Subfamily 3, Group C, Member 1
PPARG	Peroxisome Proliferator-Activated Receptor Gamma
IRF3	Interferon Regulatory Factor 3
RDS	Respiratory distress syndrome
ISSHP	International Society for the Study of Hypertension in Pregnancy
